# Epidemiology of tuberculosis among different occupational groups in Makkah region, Saudi Arabia

**DOI:** 10.1038/s41598-021-91879-9

**Published:** 2021-06-17

**Authors:** Hasan M. Semilan, Hassan A. Abugad, Husain M. Mashat, Moataza M. Abdel Wahab

**Affiliations:** 1 Ministry of Health, Makkah, Saudi Arabia; 2 Imam Abdulrahman Bin Faisal University, P.O. Box 2114, Dammam, 31451 Saudi Arabia

**Keywords:** Health occupations, Risk factors, Tuberculosis

## Abstract

Each year in Saudi Arabia, the Hajj season represents one of the world’s most significant annual mass gatherings, attracting high proportion of immigrants from different parts of the world in small crowded areas, posing a risk for Tuberculosis (TB) transmission. There is a high potential for TB contact and infection in the workplace as it is transmitted through the air. Most of the studies in Saudi Arabia assessed the TB infection among health care workers. However, the TB incidence rate among other variant occupational groups was not yet determined. This study was conducted to assess the incidence rate of tuberculosis, and determine the risk factors of TB infection among different occupational groups in the Makkah region, Saudi Arabia 2016. A cross-sectional study was carried out based on the secondary data of the patients registered in the Saudi national tuberculosis control and prevention program in 2016. Data were then organized and analyzed for age, gender, nationality, educational level, average monthly wage, average weekly working hours, and occupation of the patients. Occupations were reclassified according to the Saudi Standard Classification of Occupation (SSCO). A total of 1270 cases were included in this study, 300 (23.6%) of them were workers. The incidence rate of TB among workers in the Makkah region was 9 per 100,000 workers compared to 31 per 100,000 persons among the general population in 2016. The TB incidence rate was the highest among occupation of supporting basic engineering with 13 per 100,000 workers in 2016. The highest incidence rate of TB among occupations of supporting basic engineering could be attributed to close contact with the general population in closed spaces for long periods of time, and low socioeconomic status.

## Introduction

Tuberculosis (TB) is an infectious disease caused by *Mycobacterium tuberculosis* bacteria and most often affects the lungs (pulmonary TB) and could also affect other parts of the body (Extra-pulmonary TB). TB is transmitted from one person to another via inhalation of droplets (airborne particles) after an infected person coughs, sneezes, or speaks. The risk factors of TB infection include medical and non-medical factors. The medical factors include HIV, diabetes mellitus, immunosuppression, renal failure, and malignancy. While the non-medical factors include birth, traveling to a country with a high incidence of TB infection, working at high-risk congregate settings like prisons and shatters, and low socioeconomic status^1^.

In the United States of America, the high crystalline silica exposure had significantly increased the risk for pulmonary TB infection along with other lung diseases^2^. The exposure to TB infection increased in healthcare workers, funeral directors, construction occupations, stonemason, carpenters, and mining machine operators because of their occupations. Moreover, occupations with low socioeconomic status like butchers, automobile mechanics, clerking jobs, electrical commercial/industrial equipment repairers, and entertainers were risk factors of TB infection^3^. The low income was the most significant factor in regard to non-compliance to treatment compared with drug abuse, nonadherence to a previous treatment regimen, and history of smoking. In the subgroup of re-treatment cases, poverty is associated with a higher risk of dropout. The educational level had no association with non-compliance to treatment^4^. The health care workers’ group had a similar rate of TB as the general population. However, the rates were elevated in inhalation therapists, lower-paid health care workers, funeral directors, and farmworkers^5^. The incidence rate of TB for the foreign-born population was almost quadruple the rate for the native population of the United States^6^.

The incidence rate of TB in Saudi Arabia was 12 (10–14) per 100,000 in 2015 and was considered as a moderate burden country for TB infection^7,8^. Among immigrant workers in Al Qassim region, Saudi Arabia, females have a higher incidence rate of TB infection. The housemaid was the highest occupation with TB infection followed by daily laborers and people involved in agricultural work^9^.

## Rational

Each year in Saudi Arabia, during the Hajj season, the presence of a high proportion of immigrants in small crowded areas poses a risk for TB transmission^10^. However, the TB incidence rate was not yet determined among variant occupational groups.

This study was conducted to assess the incidence rate of tuberculosis, and determine the risk factors of TB infection among different occupational groups in the Makkah region, Saudi Arabia in 2016. The focus of this study is the epidemiology of TB among various occupational groups, rather than on the pilgrims themselves, since this could be a risk factor for TB transmission.

## Materials and method

A register-based cross-sectional study was conducted in the Makkah region. Makkah region is one of thirteen administrative regions in the Kingdom of Saudi Arabia that lies in the western part of the Arabian Peninsula. Makkah region has four directorates of health affairs which are located in Makkah city, Jeddah city, Ta'if city, and Al-Qunfudhah city. All of the TB cases from all of Makkah region cities will be reported to its corresponding directorate. Secondary data were obtained from the Ministry of Health (MOH) for the patients registered in the Saudi national tuberculosis control and prevention program through 2016 which was the most recent available data at that time.

According to the regulations of MOH, the data will not be officially released before 2 years from the date of notification to complete the follow-up data. The results mentioned in this study represent the cases registered in 2016. The data have been collected in the duration of three months from June to September 2018. Depending on the available data, demographic data: age, gender, nationality, and occupation were included in the analysis. The educational level, the average monthly wage, and the average weekly working hours of the cases were collected from the Saudi Authority of Statistics. Occupations were re-classified into main occupational groups according to Saudi Standard Classification of Occupation (SSCO) as follows: (1) occupations of services (domestic housekeepers, domestic cleaners, security guards, messengers, cooks, waiters, and hairdressers), (2) occupations of supporting basic engineering (car drivers, building construction laborers, mechanics, blacksmiths, carpenters), (3) technicians in professional, technical and humanitarian fields (nurses, medical laboratory technicians, building electricians, computer technicians, engineering technicians), (4) specialists in professional, technical and humanitarian fields (teachers, accountants, general dentists, pharmacists, specialist nurses), (5) occupations of sales (salespersons, sale representatives), (6) occupations of agriculture, animal husbandry & fishing (animal farm laborers, crop farmers), (7) occupations of clerical (secretary, administrative assistant, receptionist, post carrier, telephone switchboard operator), (8) occupations of industrial, chemical operations and food industries (machine operators, tailors), (9) lawmakers, directors and business managers (health, safety, and environment manager, sales manager), and (10) occupation of armed forces and public security^11^. The incidence rate of TB infection among each city was calculated by dividing the number of newly diagnosed cases of TB in 2016 by the total number of populations of each city in 2016. The educational level of the occupational groups was re-categorized into; high, medium, and low educational levels. The incidence rate of TB infection among each occupational group was calculated by dividing the newly diagnosed cases of TB in each occupational group in 2016 by the total number of workers of the same occupational group in 2016. The Incidence rate was calculated based on the data obtained from the Saudi General Authority for Statistics, as in the third quarter of 2016, the general population of the Makkah region was 6,276,418 persons. The total number of employed persons was 3,187,847 (50.8%) and their distribution into each occupational group, average monthly wage, and average weekly working hours are shown in (Table 1)^11^.

**Table 1:** Total number of different occupational groups with average monthly wage and average working hours in Makkah region 2016.

Main occupational groups	Total number	Average monthly wage	Average working hours
Lawmakers, Directors and Business managers	70,364	12.207	40.4
Specialists in Professional, Technical and Humanitarian Fields	291,830	14.0725	41.8
Technicians in Professional, Technical and Humanitarian Fields	288,508	6.4875	38.7
Occupations of Clerical	246,321	6.706	39.1
Occupations of Sales	432,060	4.2295	49.6
Occupations of Services	915,438	3.953	45.2
Occupations of Agriculture, Animal Husbandry & Fishing	104,799	3.165	42.7
Occupations of Industrial, Chemical Operations and Food Industries	79,852	5.2625	50.2
Occupations of Supporting Basic Engineering	758,675	4.1165	49.6
Total	3,187,847		

Cases with age under 18 or above 65 years old in both workers and non-workers were excluded. The age has been re-categorized into five groups with ten years intervals; (18–27) years, (28–37) years, (38–47) years, (48–57) years, and (58 and above) years. The categorical variable was described by numbers and percentages and the continuous variable by min, max, mean, and standard deviation. The association between the occupation status and different variables such as nationality, gender, city, and age interval were assessed by the chi-square test and fisher’s exact test. Bivariate spearman’s correlation analysis was carried out to determine the relationship between average monthly wage, average weekly working hours, and the TB infection incidence rate. The level of significance was 0.05. Data management and analysis were performed using SPSS software (SPSS Statistics).

The ethical approval was obtained from the Ethics & Research Committee of general directorate of health affairs in the Makkah region, Saudi Arabia. This study was conducted following the Declaration of Helsinki. Consent for participation was waived by the Ethics & Research Committee of general directorate of health affairs in Makkah region, Saudi Arabia.

### Ethical clearance

The study got ethical clearance from the Ethics &Research Committee of general directorate of health affairs in Makkah region, Saudi Arabia.

## Results

**Figure 1:**
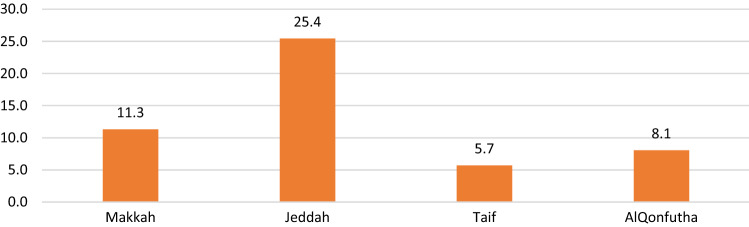
Incidence rate of TB infection per 100,000 in cities of Makkah region 2016.

**Figure 2:**
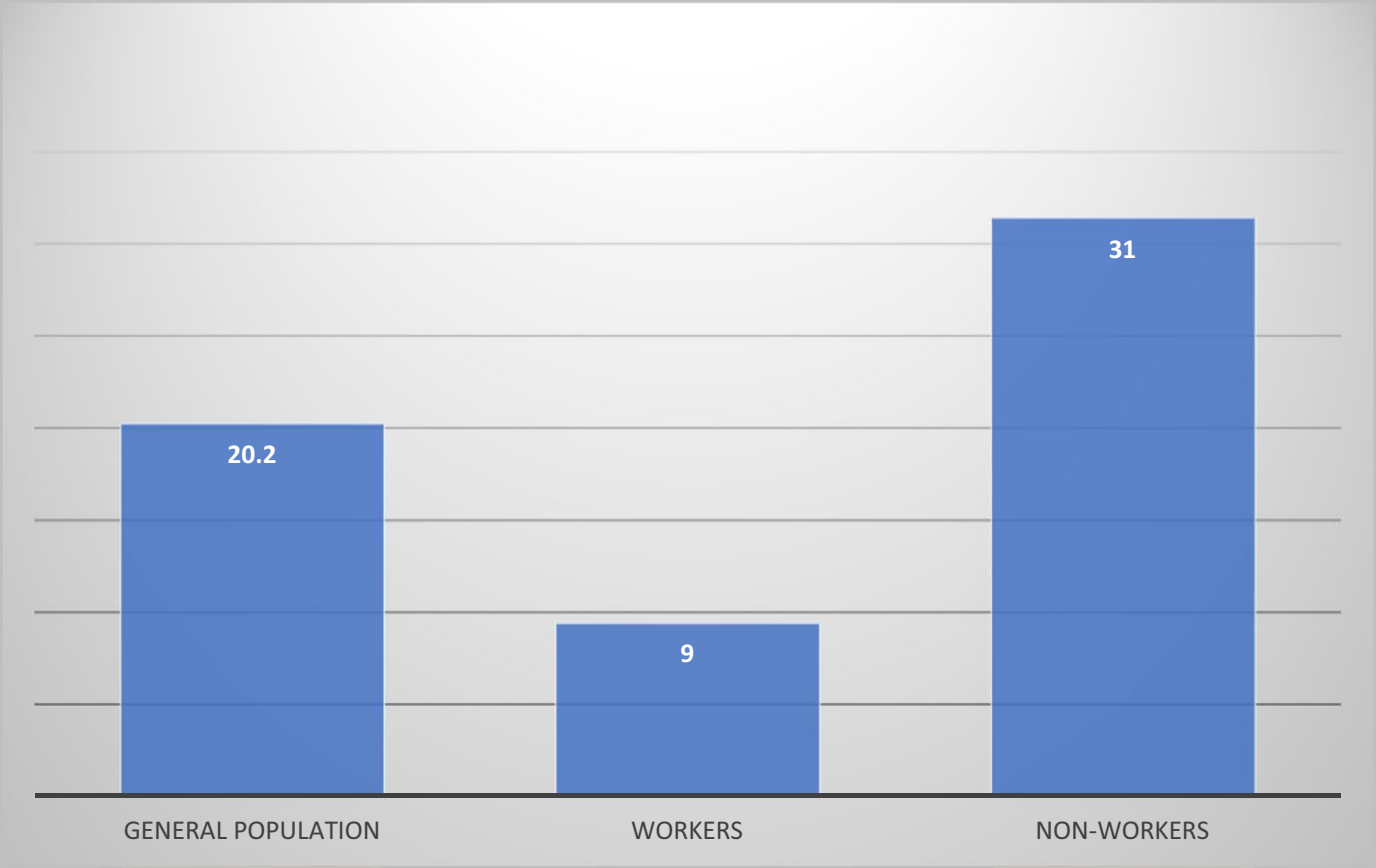
Incidence rate of TB infection per 100,000 among general population, workers and non-workers in Makkah region 2016.

The total number of confirmed TB cases registered in the national tuberculosis control and prevention program 2016 in the Makkah region after exclusion criteria were 1270 cases. Accordingly, the incidence rate of TB in the Makkah region was 20.2 per 100,000 persons among the general population. Most of the cases were in Jeddah city (76%), with an incidence rate of 25.4 per 100,000 persons (Fig. 1). The workers comprised 300 (23.6%) compared with 970 (76.4%) non-workers. The incidence rate of TB among workers was 9 per 100,000 workers compared with 31 per 100,000 non-workers (Fig. 2). Regarding nationality, 720 (63.4%) were non-Saudis compared with 416 (36.6%) Saudis. Males comprised 383 (68.2%).

Age ranged from 18 to 65 years with a mean of 35.99 ± 12.38 in workers compared with 35.29 ± 13.17 in non-workers. There was no statistically significant difference (t = 0.810, P = 0.42).

The non-Saudis were (76.3%) of the employed versus (58.7%) for non-employed, and there was a statistically significant difference (X^2^ = 29.5, P = 0.001). There were more male cases 237 (79%) compared with females 63 (21%) among the workers diagnosed with TB. A statistically significant difference was noted (X^2^ = 24.4, P = 0.001) (Table 2).

**Table 2:** Demographic distribution of TB cases according to occupational status among general population of Makkah region 2016.

		Occupational Status						X^2^	*P*
		Worker		Non-worker		Total			
		No (n = 300)	%	No (n = 970)	%	No (n = 1270)	%		
Nationality	Saudi	71	23.7	345	41.3	416	36.6	**29.5**	**0.001**
	Non-Saudi	229	76.3	491	58.7	720	63.4		
Gender	Male	237	79.0	601	64.7	838	68.2	**21.4**	**0.001**
	Female	63	21.0	328	35.3	391	31.8		
City	Makkah	59	19.7	150	16.1	209	17.0	**62.7**	**0.001**
	Jeddah*	195	65.0	740	79.6	935	76.0		
	Taif*	41	13.7	23	2.5	64	5.2		
	AlQonfutha	5	1.7	17	1.8	22	1.8		
Age interval	18–27	109	36.3	306	36.9	415	36.7	6.4	0.179
	28–37	92	30.7	219	26.4	311	27.5		
	38–47	49	16.3	116	14.0	165	14.6		
	48–57	34	11.3	122	14.7	156	13.8		
	58 and above	16	5.3	67	8.1	83	7.3		
	Mean ± SD	35.99 ± 12.38		35.29 ± 13.17		35.47 ± 12.96		t = 0.810	0.42
Site of infection	Pulmonary TB	229	76.3	847	78.3	1076	84.7	**21.4**	0.001*
	Extra-pulmonary TB	71	23.7	123	12.7	194	15.3		

*Percent of workers differed from non-workers within this category using z-test of proportion adjusted for all pairwise comparisons within a row using the Bonferroni correction.

**Figure 3:**
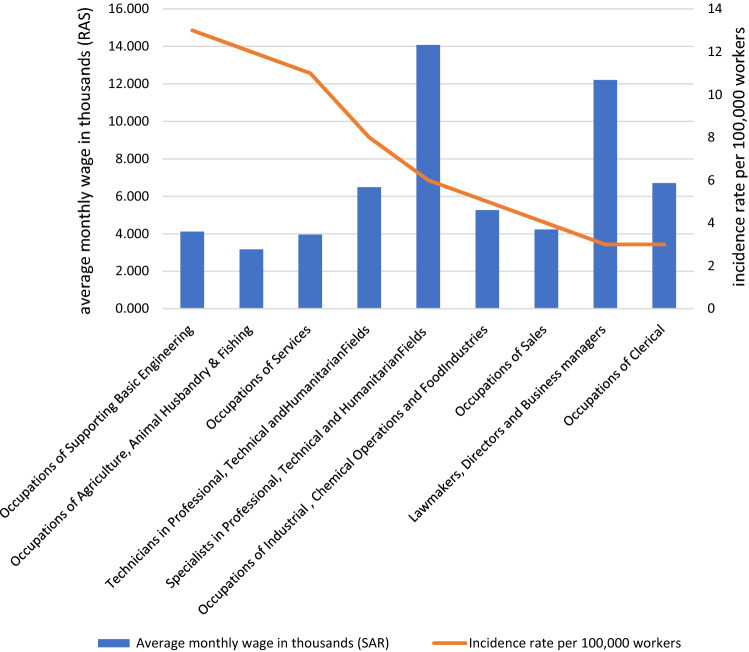
Average monthly wages and TB incidence rate among main occupational group in Makkah region 2016.

**Figure 4:**
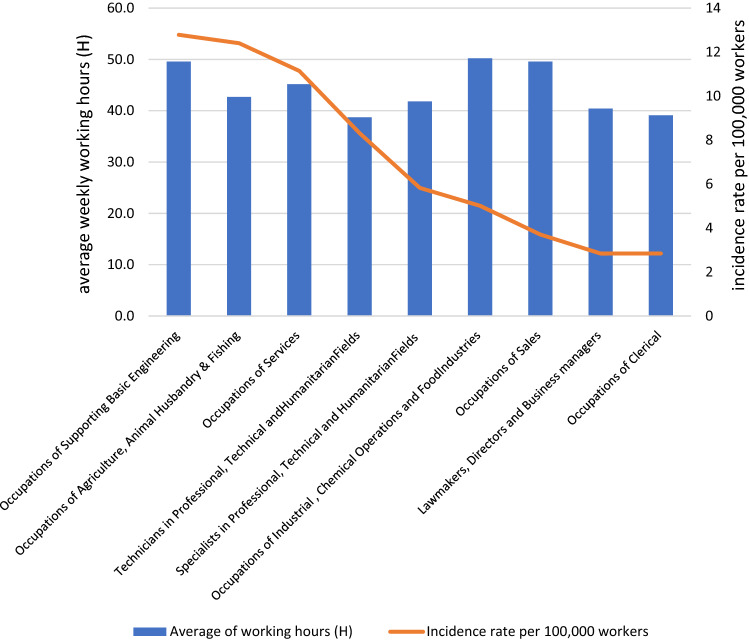
Average weekly working hours and TB incidence rate among main occupational group in Makkah region 2016.

It was observed that TB cases among workers in occupations of services represented (34%) of the cases followed by workers in occupations of supporting basic engineering (31.7%), and occupations of technicians in professional, technical, and humanitarian fields (8%) as shown in (Table 3). Non-Saudis outnumbered Saudis in every occupational category except the armed forces and public security, which are reserved exclusively for Saudis, and there was a statistically significant difference (X^2^ = 102.96, P = 0.001) (Table 3). The highest TB incidence rate was documented among workers in occupations of supporting basic engineering 13 per 100,000 workers, followed by workers in occupations of agriculture, animal husbandry, fishing, and workers in occupations of services with 12,11 per 100,000 workers, respectively. The average monthly wage for occupations and the incidence rate of TB among occupations are shown in (Fig. 3), and there was a statistically significant intermediate negative correlation between them (r = -0.678, P = 0.045) (Table 4). Regarding the average working hours, there was no statistically significant correlation with TB incidence (r = 0.277, P = 0.470) (Fig. 4) (Table 4). Regarding the educational level, there was no correlation between the incidence rate of TB infection and high educational level (HEL). There was an inverse association between both medium educational level (MEL) and low educational level (LEL) and the incidence rate of TB. No statistically significant difference was evident (Table 4). The case fatality rate from TB among workers was (2.7%) compared with (3.6%) among non-workers, though there was no statistically significant difference (X^2^ = 0.23, P = 0.631). (not shown in the tables).

**Table 3:** Comparing the Nationality among main occupational group of TB cases among workers in Makkah region 2016.

		Nationality					
		Saudi		Non-Saudi		Total	
		No (n = 71)	%	No (n = 229)	%	No (n = 300)	%
Main occupational group	Occupations of Services	22	21.6	80	78.4	102	34.0*
	Occupations of Supporting Basic Engineering	5	5.3	90	94.7	95	31.7*
	Technicians in Professional, Technical and Humanitarian Fields	7	29.2	17	70.8	24	8.0*
	Occupation of Armed Forces and Public Security	20	100	0	0	20	6.7
	Specialists in Professional, Technical and Humanitarian Fields	8	47.1	9	52.9	17	5.7
	Occupations of Sales	2	12.5	14	87.5	16	5.3
	Occupations of Agriculture, Animal Husbandry & Fishing	1	7.7	12	92.3	13	4.3
	Occupations of Clerical	5	71.4	2	28.6	7	2.3
	Occupations of Industrial , Chemical Operations and Food Industries	0	0	4	100	4	1.3
	Lawmakers, Directors and Business managers	1	50.0	1	50.0	2	0.7
	X^2^	101.91					
	*P*	0.001

**Table 4:** Correlation between incidence rate of TB with average monthly wage, average weekly working hours and educational level among workers of Makkah region 2016.

Correlation with incidence rate of TB	Average monthly wage	Average weekly working hours	Educational level		
			Low	Medium	High
*r*_*s*_	−.678*	.277	−.586	−.310	−.538
*p*	.045	.470	.097	.417	.135

The extra-pulmonary TB represents (15.3%) of the studied cases, with a higher percentage among workers (23.7%) compared with (12.7%) among non-workers. A statistically significant difference was evident (X^2^ = 21.4, P = 0.001) (Table 2). However, there was no statistically significant difference in the distribution of extra-pulmonary TB among different occupational groups (X^2^ = 11.72, P = 0.23).

## Discussion

Makkah region has reported the highest incidence rate of tuberculosis in Saudi Arabia through the period between 2005 to 2012, with an estimate of 25.13 per 100,000 persons. This could be attributed to the Hajj season, with high numbers of immigrants in this region, and Saudi Arabia's large workforce in general, all of which make the area favorable to TB transmission^10,12,13^.

The incidence rate of TB in Makkah region showed a perceptible decline in 2016 compared with the period from 2005 to 2012^13^. Most of the TB cases were in Jeddah city. The incidence rate of TB among workers was noticeably lower than the general population in Makkah region in 2016. Regardless of occupational status, the proportion of male cases was greater than females', which is consistent with the consensus that male gender is a risk factor for TB infection compared with females^14^. Furthermore, the greater incidence of TB cases among male workers in Saudi Arabia could be attributed to the country's male-dominated workforce^11^. Regarding nationality, non-Saudi workers had a higher TB incidence rate, which is a reflection of the general population incidence rate where the non-Saudi had a higher TB incidence rate. Moreover, most of the workers came from high TB burden countries and they comprise (63.3%) of the total working population in the Makkah region 2016^11,15^. Other risk factors of TB infection are the low socioeconomic status which is noticeable among non-Saudi workers^16^. Furthermore, the increased likelihood of avoiding medical treatment owing to fear of deportation or job loss as a consequence of a diagnosis of such an infection may contribute to an increase in the incidence rate.

However, we found that there is a statistically significant difference in the numbers of TB cases among the different occupational groups. The highest proportion of TB cases was among the workers in occupations of services followed by workers in occupations of supporting basic engineering. This was reasonable as these occupational groups acquire half of the working population in the Makkah region (29%, 24%), respectively^11^.

The disparity between the number of cases and the incidence rate suggests that the nature of the occupation could be a risk factor for TB infection.

The occupations of supporting basic engineering had the highest incidence rate of TB. This could be due to close contact with other people in closed spaces for long periods of time or long working hours such as in car drivers^17^. Certain occupations, such as building construction laborers, may encounter exposure to silica dust, which may cause silicosis, and silico-tuberculosis. Moreover, the low socioeconomic (low average monthly wage) status may compel the workers to live in small crowded rooms, which contribute to the increase of the incidence rate of TB^2,3,18^.

Occupations of agriculture, animal husbandry, and fishing reported an almost identical incidence rate of TB. This could be attributed to the increased risk of TB (Bovine Tuberculosis) infection from infected animals, such as in animal farm laborers and crop farmers^5,19^. The occupations of services such as domestic housekeepers and domestic cleaners, had the third-highest TB incidence rate. This could be due to close contact with other people or the nature of their occupation, which necessitates constant presence around people. Additionally, they commonly originate from countries with high TB burdens^9^. Other occupations in this group work in closed, crowded areas, and might be exposed to active TB cases in accommodation complexes or prisons which is considered as an occupational risk for TB infection such as security guards^1,20^. Messengers who are also part of the group may be exposed to infected people as a result of their occupation, which requires them to visit a variety of places and interact with different individuals, which might be a risk factor for TB infection. Consistent with several previous studies, our findings suggested that occupations with low socioeconomic status were a risk factor of TB infection^3,4^.

Despite the statistically significant difference of extra-pulmonary TB between workers and non-workers, the role of other confounding factors can not be excluded, and this might be the cause of this finding.

There was no association between the incidence rate of TB and the level of education among different occupational groups which is consistent with results from previous studies^4^.

A key strength of this study lies within the fact that it includes all the TB-positive cases in the working-age (18–65). Additionally, it is the first study conducted to assess the incidence rate of TB among all the occupational groups.

The encountered limitations of this study were deficient detailed information regarding the lifestyle and socioeconomic condition, and the total number of workers for each occupational group in each city of the Makkah region were not available. Also, the occupations were not under any classification model. Moreover, the mortality rate among each occupational group and exposure history to occupational risk factors such as silica were not available.

## Conclusion

The occupations of supporting basic engineering including (car drivers, building construction laborers, mechanics, blacksmiths, carpenters) had the highest incidence of TB infection, which could be related to the close contacts with the general population in closed spaces for a long period of time, and they might be exposed to silica dust which may cause silicosis and silico-tuberculosis. The occupations with low socioeconomic status (low average monthly wage) could be considered as a risk factor for TB infection. Moreover, the lack of health promotion and occupational health services could be a precipitating factor for TB infection along with other diseases.

## Recommendations

Preventive and controlling measures should be directed toward the working population included in occupations of supporting basic engineering groups, occupations of agriculture, animal husbandry & fishing, and occupations of services.

Basic occupational health services including health promotion in the workplaces and periodic examination should be implemented especially among the most affected occupational groups.

Further research with more detailed socioeconomic, educational level, and lifestyle data should be conducted with a history of exposure to occupational risk factors to properly address the occupational risk factors associated with TB infection among working groups.
